# Primary care team and its association with quality of care for people with multimorbidity: a systematic review

**DOI:** 10.1186/s12875-023-01968-z

**Published:** 2023-01-19

**Authors:** Mingyue Li, Haoqing Tang, Xiaoyun Liu

**Affiliations:** 1 Department of Health Policy and Management, School of Public Health, Peking University, Beijing, China; 2 China Center for Health Development Studies, Peking University, 38 Xue Yuan Road, Beijing, 100191 China

**Keywords:** Primary care, Teams, Chronic disease management

## Abstract

**Background:**

Multimorbidity is posing an enormous burden to health systems, especially for primary healthcare system. While primary care teams (PCTs) are believed to have potentials to improve quality of primary health care (PHC), less is known about their impact on the quality of care for people with multimorbidity. We assessed the characteristics of PCTs and their impact on the quality of care for people with multimorbidity and the mechanisms.

**Methods:**

We searched PubMed, MEDLINE, EMBASE, ProQuest for published studies from January 2000 to October 2021 for studies in English. Following through PRISMA guidelines, two reviewers independently abstracted data and reconciled by consensus with a third reviewer. Titles, abstracts, and full texts were evaluated to identify relevant studies. Studies were categorized by types of interventions, the impact of interventions on outcome measures, and mechanisms of interventions.

**Results:**

Seventeen studies (13 RCT, 3 cohort studies, and 1 non-randomized trial) were identified. PCTs were summarized into three types—upward PCTs, downward PCTs and traditional PCTs according to the skill mix. The upward PCTs included primary care workers and specialists from upper-level hospitals, downward PCTs involving primary care workers and lay health workers, and traditional PCTs involving physicians and care managers. PCTs improved patients’ mental and psychological health outcomes greatly, and also improved patients’ perceptions towards care including satisfaction with care, sense of improvement, and patient-centeredness. PCTs also improved the process of care and changed providers’ behaviors. However, PCTs showed mixed effects on clinical outcome measures.

**Conclusions:**

PCTs have improved mental and psychological health outcomes, the process of care, patients’ care experiences, and satisfaction towards care for patients with multimorbidity. The effect of PCTs on clinical outcomes and changes in patient behaviors need to be further explored.

**Supplementary Information:**

The online version contains supplementary material available at 10.1186/s12875-023-01968-z.

## Introduction

Chronic diseases have become an enormous burden to society and global health systems [1]. People with chronic conditions are likely to have more than one disease, which is referred to as multimorbidity. Multimorbidity is becoming increasingly common both in high-income countries and low-and-middle income countries. One-fourth of people in the United States and the United Kingdom have multimorbidity, and at least two-thirds among adults aged 65 and over [2, 3]. In China, more than 40% of adults aged 60 and over in the mainland have multimorbidity [4]. People with multimorbidity are likely to have worse health outcomes and increased mortality [5], calling for greater recognition of its impact on individuals affected [2, 6]. Multimorbidity challenges the traditional healthcare system that focuses on managing individual diseases [7]. A people-centered and integrated healthcare delivery system has been promoted increasingly to enhance quality and meet the needs of people with multimorbidity [8].

Strengthening primary health care (PHC) system has been proposed as the key to improve health outcomes for people with multimorbidity due to their comprehensive healthcare needs and prevention services needs that episodic specialized care cannot meet [9]. PHC, with the characteristics of continuity, comprehensiveness and coordination, is associated with higher value care at the whole-person level, better health, greater equity, lower costs at the level of populations [10, 11].

Primary care teams (PCTs) incorporating interdisciplinary collaboration have proved potential to deal with complex patients [12, 13]. Many developed countries are seeing PCTs as new directions of healthcare reform, such as the patient-centered medical home in US [14], the primary care home in UK, and the family health team model in Canada [15]. These initiatives seek to improve access to care of high quality, transition care from emphasizing volume to value and increase effectiveness through task-shifting [15, 16]. However, there are gaps of their implementation and organizational arrangements, especially concerning patients with complex conditions with more healthcare need.

This systematic review aims to synthesize existing evidence, identify and evaluate the impact of interventions in PCTs designed to improve care among people with multiple chronic conditions on quality of care. Based on this objective, we try to answer the following research questions:

1. What are the characteristics of PCTs designed to improve care for people with multimorbidity?
2. What are the impacts of PCTs on the quality of care among people with multimorbidity in primary health care?
3. What are the mechanisms by which PCTs influence the quality of care among people with multimorbidity in primary health care?

The findings of this paper can inform the development of PCTs and enhance better management of people with multimorbidity.

## Methods

This systematic review was performed according to the PRISMA guidelines. Our review is registered with the PROSPERO database (PROSPERO CRD42021284242). No protocol was published. A comprehensive search was conducted of the online databases: PubMed, MEDLINE, EMBASE, ProQuest for studies in English using indexed and free text words for the past twenty years (January 2000-October 2021). The keywords and MeSH terms used were “multimorbidity” “patient care teams” and “primary health care”. The step-by-step search strategies for the four databases are presented in the supplementary file 1. Reference lists of retrieved studies and systematic reviews were also examined for relevant studies. Companion documents were also searched to obtain more information on the detailed PCTs interventions, including protocols, supplementary files for each publication, website pages, etc.

### Definitions of terms and eligibility criteria

#### Types of population

The participants are people or populations with multimorbidity receiving care in primary or community care settings. The most widely used definition for multimorbidity is the co-occurrence of two or more chronic health conditions in the same individual [17, 18]. Multimorbidity is often used interchangeably with comorbidity, which refers to a specific pair of diseases beyond the index disease under study, so we also included studies using “comorbidity” in this review. In this paper, we adopted “multimorbidity” according to the widely-used definition by Van den Akker and colleagues [8, 19, 20]. To increase the homogeneity of the sample and comparability of PCTs characteristics across studies, the homeless population, children, pregnant women, the oldest-old population were excluded. Studies that focused on one single disease were also excluded.

#### Types of interventions

There is no consistent definition for PCTs currently. Basically, PCTs refer to a group of health-related workers working together to provide health services. Chevillard and Mousquès defined PCTs as “multiprofessional group practices with at least two GPs and one paramedic, delivering primary care and services based on cooperation and coordination” [21]. As more health workers from other disciplines join in PCTs, a growing change to the definition of PCTs has sparked renewed interests. Wranik et al. narrowed the definition of PCTs down to interprofessional PCTs (IPPC team) – “consisting of healthcare providers from different disciplines working together to address the health needs of populations through the creation of comprehensive care options, increased continuity, and coordination of care” [13]. The origin of PCTs dated back to 1950s in the UK, when general practitioners (GPs) in the primary health system usually worked single-handed back then. The concept of PCTs first appeared in the UK with the arrival of NHS in the 1950s, when the College of Practitioners encouraged a team approach in primary health care [22], followed by Finland and Netherlands [23]. A group practice model was gradually formed. PCTs only included a GP and a nurse at first, but it has expanded over the years, incorporating other healthcare professionals, managers [24], pharmacists [25], social workers [26], mental health workers [27], nutritionists [28], etc.

PCTs have often been used interchangeably with “primary healthcare teams” or “primary health teams”. The healthcare services provided by the primary care teams are “team-based care”, which is also often used in studies. We also included these terms in the review process.

#### Types of outcomes

Currently, there is no consensus in understanding of “quality of care” and disagreements remain about what it encompasses. The classic definition for quality of care was developed by Donabedian in 1966. He built on the concept of “input–process–output” used in industrial manufacturing, and proposed the triad of “structure-process-outcome” for quality of care [29]. This definition has been widely accepted and used in describing and evaluation quality of care [30].

Vast literature discussed and tried to define quality of care in various contexts after Donabedian’s work. In 1984, the American Medical Association defined care of high-quality as care “which consistently contributes to improvement or maintenance of the quality and/or duration of life” [31]. The association also specified eight essential elements of quality, emphasizing health outcomes, disease prevention, health promotion, timeliness, patient participation, the scientific basis of medicine, attention to patients’ psychological conditions, efficient use of resources, and sufficiently documented medical records. In 1990, the Institute of Medicine (IoM) defined quality as “the degree to which health care services for individuals and populations increase the likelihood of desired outcomes and are consistent with current professional knowledge” [32]. The definition of IoM is also widely acknowledged for its emphasis on health of populations, patients’ comprehensive wellbeing and the limitation of medicine.

Based on the most widely used definitions for quality of care above, we focused on the quality of process and outcome, which included 1) clinical outcome measures of patient health outcomes such as physical or psychological outcomes, 2) subjective measures of health outcomes like patient-reported outcomes and experiences, 3) measures of changes in patient health behaviors, 4) process measures like prescription patterns.

### Data extraction and quality appraisal

Two reviewers (ML and HT) independently abstracted data using the eligibility criteria and discussed disagreements. Before the review process, a screening exercise was performed for two reviewers to make sure that the evaluation process was reliable. Titles and abstracts were first evaluated, and ineligible studies were excluded. Full texts were evaluated of the selected studies afterward. Discrepancies at any time were solved by a third reviewer (XL) and within the research team. The following information was extracted using a standardized data extraction tool: author (year) county, title, study design, study aims, settings, sample size, disease types, inclusion criteria, exclusion criteria, team composition, roles of team members, intervention training, communication and supervision, control group, study phase, outcomes, results. The synthesis was conducted in Microsoft Excel 2019. The review methodology followed through PRISMA guidelines for systematic reviews. For studies of RCT, risk of bias was reported based on the Cochrane criteria (supplementary file 2). The Mixed Methods Appraisal Tool (MMAT) was used to assess the methodological quality for each study, and the quality of the studies was rated at a five-point scale (supplementary file 2) [33].

## Results

### Study characteristics

**Fig. 1:**
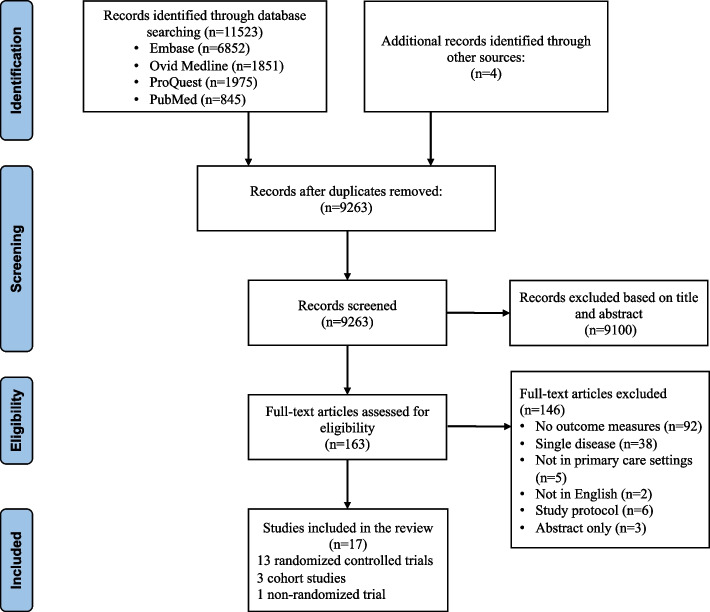
PRISMA flow diagram of the studies selection process

Overall, 9263 unique records were identified, of which 9100 records were excluded based on title and abstract screening. One hundred sixty-three records were assessed in full-text for eligibility, among which 92 were excluded because they did not contain outcome measures, 38 were excluded because they focused on a single disease, 5 were excluded because they were not in primary care settings, 2 were excluded for language, 6 were excluded because only protocols were available, and 3 were excluded because of abstracts only (Fig. 1). Therefore, seventeen studies (13 RCTs, 3 cohort studies, and 1 non-randomized trial) were included. Among these studies, seven were conducted in the United States, 4 in the United Kingdom, 1 in Germany, 1 in Taiwan China, 2 in South Africa, 1 in Australia, and 1 in Spain. The sample size of the included studies ranged from 142 to 5 337 377. Eleven studies studied depression comorbid with other diseases, such as hypertension, diabetes, or coronary heart disease, 5 studies studied other chronic diseases, 1 studied multimorbidity in general. Sixteen models of primary care teams were identified in the included studies, among which two studies assessed the same intervention at different times. All studies were based on complex and multifaceted interventions or policies, and no study linked quality change in a single intervention component (Table 1).

**Table 1:** Characteristics of the studies

Literature	Design	Study aims	Settings	Sample size	Disease type	Overall score
Aragonès, et al. 2019, Spain [34]	RCT	To assess the effectiveness of a collaborative care program designed for the management of major depression and chronic musculoskeletal pain	urban primary care centers	I: 167 C: 161	major depression and chronic musculoskeletal pain	***** 100%
Chen, et al. 2010, USA [35]	Cohort study	To evaluate the impact of the Teamlet Model on care of patients with diabetes and/or hypertension in a primary care residency practice	family medicine teaching clinic	I: 146 C: 395	diabetes and/or hypertension	***** 100%
Coventry, et al. 2015, UK [36]	RCT	To test the effectiveness of an integrated collaborative care model for people with depression and long-term physical conditions	general practices	I: 191 C: 196	depression comorbid with diabetes or cardiovascular disease	**** 80%
Freund, et al. 2016, Germany [37]	RCT	To determine whether protocol-based care management delivered by medical assistants improves care in patients at high risk for future hospitalization in primary care	primary care practices	2076	patients with type 2 diabetes, chronic obstructive pulmonary disease, or chronic heart failure	**** 80%
Jan, et al. 2021, China [38]	Cohort study	To explore the impact of Taiwan’s Family Practice Integrated Care Project (FPICP) on hospitalization	clinics and community hospitals	I: 2,316,114 C: 3,021,263	COPD/asthma, diabetes and its complications, heart failure	**** 80%
Katon, et al. 2004, USA [39]	RCT	To determine whether enhancing quality of care for depression improves both depression and diabetes outcomes in patients with depression and diabetes	clinics	I: 164 C: 165	diabetes mellitus and major depression	***** 100%
Katon, et al. 2010, USA [40]	RCT	To determine whether coordinated care management of multiple conditions improves disease control in these patients	clinics	I: 106 C: 108	diabetes, coronary heart disease or both and co-existing depression	***** 100%
Lin, et al. 2012, USA [41]	RCT	To assess patient and physician behaviors (medication adherence, self-monitoring, and treatment adjustment) in achieving better outcomes for diabetes, coronary heart disease, and depression	clinics in Group Health Cooperative	I: 90 C: 91	multiple conditions, depression, and poorly controlled diabetes or coronary heart disease	***** 100%
Morgan, et al. 2013, Australia [42]	RCT	To determine the effectiveness of collaborative care in reducing depression in primary care patients with diabetes or heart disease using practice nurses as case managers	general practices	I: 206 C: 194	depression and type 2 diabetes, coronary heart disease or both	** 40%
Petersen, et al. 2019, South Africa [43]	Cohort study	To evaluate a task shared integrated collaborative care package of care for chronic patients with co-existing depressive and AUD symptoms	primary care facilities	1310	Chronic patients with co-existing depressive and AUD symptoms	**** 80%
Petersen, et al. 2021, South Africa [44]	RCT	To test the hypothesis that the Program for Improving Mental health care (PRIME) collaborative model would be more effective than care as usual for patients with hypertension and comorbid depressive symptoms and would improve both depressive symptoms and blood pressure control	clinics	I: 441 C: 484	hypertension and depression	**** 80%
Salisbury, et al. 2018, UK [45]	RCT	To assess the effectiveness of a patient-centered, so-called 3D approach for patients with multimorbidity in improving their health-related quality of life	general practices	I: 797 C: 749	multimorbidity in general	**** 80%
Sharpe, et al. 2014, UK [46]	RCT	To compare the effectiveness of an integrated treatment program for major depression in patients with cancer with usual care	cancer centers, associated primary clinics	I: 253 C: 247	cancer and depression	***** 100%
Towfighi, et al. 2021, USA [47]	RCT	To determine if a chronic care model–based, community health worker, APC, and physician team intervention improves risk factor control after stroke in a safety-net setting	public safety-net healthcare system hospitals	I: 241 C: 246	ischemic attack, ischemic stroke, or intracerebral hemorrhage; and hypertension	***** 100%
Walker, et al. 2014, UK [48]	RCT	To assess the efficacy of an integrated treatment program for major depression in patients with lung cancer compared with usual care	cancer centers, associated clinics	I: 68 C:74	lung cancer and depression	***** 100%
Wolff, et al. 2021, USA [49]	Non-randomized trial	To assess the effect of integrated care on physical and mental health outcomes among low-income Latino participants on the US-Mexico border	clinics, community health centers, mental health organizations	I: 1559 C: 1396	diabetes, depression, hypertension or obesity	*** 60%
Wood, et al. 2008, USA [50]	RCT	To investigate whether a nurse-coordinated multidisciplinary, family-based preventive cardiology program could improve standards of preventive care in routine clinical practice	hospitals, general practices	I: 2778 C: 2627	CAD or were on treatment with antihypertensive or lipid-lowering drugs	*** 60%

*I &C* Intervention group, control group, *AUD* Alcohol use disorder, *CAD* Coronary heart disease, *APC* Advanced practice nurse, including nurse practitioners or physician assistants, *3D* Based on dimensions of health, depression, and drugs

### Primary care teams

**Fig. 2:**
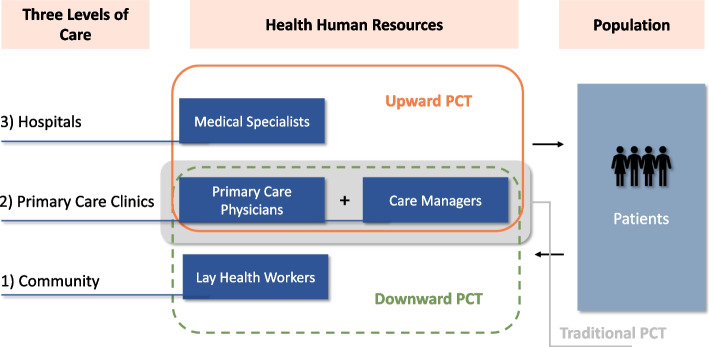
Classifications of primary care teams

The PCTs in the included studies were summarized below. A detailed description of the interventions was presented in table S2 (supplementary file 3). Basically, PCTs involved in the included studies can be broadly summarized into three categories based on the skill mix of the team: 1) upward collaborative PCTs involving primary care workers and specialists from upper-level hospitals [51], 2) downward PCTs involving primary care workers and lay health workers such as community health workers, and 3) traditional PCTs involving primary care physicians and care managers (Fig. 2). Some PCTs, such as TEAMcare, also emphasized the roles of patients in the team and their collaborative work with other team members [41].

The upward collaborative PCTs included 5 models in 7 articles [40, 41, 44, 46, 48, 49, 52]. In this model, health workers from primary care clinics worked together with hospital specialists, such as psychiatrists, psychologists, internists, etc. The specialists trained primary health workers with essential knowledge and skills, supervised, and gave recommendations for the care process. In the TEAMcare study, a weekly meeting was arranged among nurses, primary care physicians, a psychiatrist, and a psychologist to review new cases and patient progress [40, 41]. In the multi-site study on the US-Mexico border, the integration and collaboration among multiple healthcare providers were emphasized by introducing warm-handoffs, transportation support, and a transitional nurse [49]. However, this study was piloted in various places, and the interventions were tailored to the local organizations' context, setting, and population. Multiple professionals emerged in different places, including physicians and nurses from different primary care clinics, community health workers, or specialists from local hospitals.

The downward collaborative PCTs included 2 models in 2 articles [43, 47]. In this model, primary care workers worked collaboratively with lay workers from the community, such as community health workers or lay counselors. A collaborative care model for patients comorbid with mental disorders and chronic conditions in South Africa was composed of primary care nurses, GPs, and lay counsellors [43]. The primary care nurses were trained to provide person-centered care and supplementary mental health training. The lay counselors were trained and delivered group-based counseling services drawing on cognitive behavioral therapy techniques and also referred patients for further counseling. In the SUCCEED trial targeting patients with transient ischemic attack with multimorbidity, the community health worker (CHW) was the core member of the team who served as a liaison between the patient and the health system [47]. They reinforced and enhanced health literacy, risk factor control, self-management, and lifestyle changes for patients. The CHW and the physicians meet frequently to discuss patients’ progress and needs.

The traditional PCTs included 8 models in 8 articles [34–38, 42, 45, 50]. In this model, primary care physicians and nurses/medical assistants (care managers) were the main members. Basically, the care managers had close contact with patients to help them manage their conditions within daily life and build a bridge between patients and the healthcare system. In the DROP program, the care managers regularly monitored patients by phone, provided therapeutic advice, and reminded them about upcoming appointments. They also delivered a cognitive-behavioral psychoeducational program for patients, promoting awareness and self-management [34]. The care managers and primary care physicians were loosely connected by information system. A computerized decision support system, integrated in the clinical electronic medical record system in primary care clinics, can generate general recommendations to support physicians’ decision-making. Care managers annotated these suggestions in the clinical record of each patient as delivery. The Teamlet model was also a typical one. It was a small team composed of three persons—a primary care physician and two health coaches (care managers) who were trained from medical assistants or health workers [35]. Under this small team, a clinical encounter was extended to four parts: a pre-visit by the coach, a visit by the physician together with the coach, a post-visit by the coach, and between-visit care by the coach. The coach provided guidance on managing their diseases and emotional support to patients. They also monitored patients’ progress, solved their problems, and navigated the healthcare system.

### The impact of PCTs on quality of care

The impact of PCTs on quality of care can be summarized as follows: first, PCTs improved patients’ mental and psychological health outcomes significantly. The majority of depression-related measurements like SCL-90, SCL-20, PHQ-9 or GAD-7, showed significant improvements; Second, PCTs improved patients’ perceptions towards care. Patients’ satisfaction with care, perceptions of improvement, and patient-centeredness all showed significant improvements; Third, PCTs have changed the process of care. Although providing examinations showed mixed results, patients had more consultations with PCTs, and the continuity of care also increased. More medication adjustment made by physicians was also observed. Lastly, PCTs showed mixed effects on clinical outcome measures. The results of changes in BP, HbA1c, or LDL were mixed.

#### Clinical outcome measures

All the 17 studies reported clinical outcome measures (Table 2). The effect of PCTs on improving blood pressure (BP), hemoglobin A1c (HbA1c), low-density lipoprotein cholesterol levels (LDL) were mixed. Two studies reported statistically significant improvements in BP [40, 50], but the other four studies reported no significant improvements [35, 44, 47, 49]. Katon et al. found that patients systolic BP decreased by 5.1 mmHg (*P* < 0.001) compared with the control group [40], and Wood et al. found that 65% of patients attained the goal of reaching less than 140/90 mmHg compared with 55% in the control group (95%CI = 0.6–20.2, *P* = 0.04) [50]. Two studies reported significant improvement in HbA1c [40, 49], and three studies did not find significant improvements [35, 47, 52]. One study found significant improvement in BMI [35], two studies found no significant improvements [44, 47], and one study found no significant improvement in waist circumference [44]. Two studies reported significant improvements in LDL [35, 40], and two studies reported no significant improvements [47, 50].

**Table 2:** Study phase and results of the included studies

Studies	Study phase	Results
Aragonès, et al. 2019, Spain [34]	12 months	Clinical outcome measures: Severity of depression score, 0.23 points lower (1.11 vs. 1.34; 95%CI -0.42–0.04); Response rate to antidepressant treatment,18.9% higher (39.6% vs. 20.7%; OR = 2.74; 95% CI 1.12–6.67); Remission rate for depression, 9.0% higher (20.1% vs. 11.1%; OR = 2.13; 95%CI 0.94–4.85); Pain severity, pain response rate: no significant difference Patient-reported measures, behavior measures, process measures: none
Chen, et al. 2010, USA [35]	12 months	Clinical outcome measures: BMI: + 85.0% compared with baseline (*P* < 0.001) Patient-reported measures, behaviors measures: none Process measures: Self-management plan made, + 35.6% compared with baseline (*P* < 0.001); Assessing smoking status, + 82.8% compared with baseline (*P* < 0.001); Testing for LDL, -5.8% lower compared with controls (*P* < 0.001)
Freund, et al. 2016, Germany [37]	24 months	Clinical outcome measures: Quality of life, 1.16 (95% CI 0.24–2.08) on SF-12 physical component and 1.68 (95% CI 0.60–2.77) on SF-12; Mental component improved significantly at 24 months; General health, EQ-5D, 0.03 (95%CI,0.00–0.05) improved significantly at 24 months; All-cause hospitalizations, no significant difference Patient-reported measures, behaviors measures, process measures: none
Coventry, et al. 2015, UK [36]	4 months	Clinical outcome measures: Mean depressive scores, 0.23 SCL-D13 points lower (95% CI -0.41–0.05); Anxiety, a reduction of 1.45 points (95%CI -2.45–0.56) on the GAD-7; Patient-reported measures: Satisfaction with care, more patient centered, total score on PACIC (2.37 (SD 1.0) vs 1.98 (SD 0.9); More satisfied with care, total score on CSQ (2.90 (SD 0.6) vs 2.62 (SD 0.6)); Self-efficacy, illness-perceptions, disability, social support, no significant difference Behaviors measures: Self-management, better in self-management Process measures: none
Jan, et al. 2021, China [38]	1 year	Clinical outcome measures: Hospitalization, lower for COPD/asthma (OR = 0.91, 95% CI 0.87–0.94, in 2015) and for diabetes or its complications (OR = 0.87, 95% CI 0.83- 0.92, in 2015) Patient-reported measures, behaviors measures, process measures: none
Katon, et al. 2004, USA [52]	12 months	Clinical outcome measures: HbA1c levels, no significant difference; Depression severity, less severity over time (*P* = 0.004);Adequacy of dosage of antidepressant medication treatment, greater improvement in the first 6-month period (OR = 4.15, 95%CI 2.28–7.55) and the second 6-month period (OR = 2.90, 95%CI 1.69–4.98) Patient-reported measures: Satisfaction with care, higher at 6 months (OR = 2.01, 95% CI 1.18–3.43) and 12 months (OR = 2.88, 95% CI 1.67- 4.97); Patient-rated global improvement, higher at 6 months (intervention 69.4% vs usual care 39.3%, OR = 3.50, 95%CI 2.16–5.68) and 12 months (intervention 71.9% vs usual care 42.3%, OR = 3.50, 95% CI 2.14–5.72) Behaviors measures, process measures: none
Katon, et al. 2010, USA [40]	12 months	Clinical outcome measures: Glycated hemoglobin levels (difference, 0.58%); LDL cholesterol levels (difference, 6.9 mg per deciliter (0.2 mmol per liter)); Systolic BP (difference, 5.1 mm Hg); SCL-20 depression scores (difference, 0.40 points) (*P* < 0.001); Better quality of life (*P* < 0.001) Patient-reported measures: Greater satisfaction with care for diabetes, coronary heart disease, or both (*P* < 0.001) and with care for depression (*P* < 0.001) Behaviors measures: none Process measures: More likely to have adjustments of insulin (P = 0.006), antihypertensive medications (*P* < 0.001), antidepressant medications (*P* < 0.001)
Lin, et al. 2012, USA [41]	12 months	Clinical outcome measures, patient-reported measures: none Behaviors measures: No significant difference of medication adherence at 12 months Process measures: Monitoring BP and glucose more frequently, RR = 1.28 (*P* = 0.006); Antihypertensive medications initiation and adjustment, RR = 1.86, (*P* < 0.001); Insulin initiation and adjustment, RR = 2.97, (*P* < 0.001); Anti-depressants initiation and adjustment, RR = 6.20, (*P* < 0.001)
Morgan, et al. 2013, Australia [42]	12 months	Clinical outcome measures: Mean depression scores, after 6 months of intervention for patients with moderate-to-severe depression decreased by 5.7 ± 1.3 compared with 4.3 ± 1.2 in control, a significant (*p* = 0.012) difference Patient-reported measures: none Behaviors measures: Exercise, increased by 19% (not change in the control practice); Visits to mental health workers, increased by 17% (decreased by 3%) Process measures: Referrals to exercise program increased by 16% (decreased by 5%); Referrals to mental health workers increased by 7% (increased 12%)
Petersen, et al. 2019, South Africa [43]	12 months	Patient-reported measures: PHQ-9: patients with depressive symptoms having more than a 50% reduction in PHQ-9 scores were greater in the treatment group (*n* = 69, 55.2%) compared to the comparison group (*n* = 49, 23.4%) at 3 months (RR = 2.10, *p* < 0.001); 12 months follow-up (intervention: *n* = 57, 47.9%, comparison, *n* = 60, 30.8%, RR = 1.52, *p* = 0.006); Remission (PHQ-9 ≤ 5) was greater in the intervention group (*n* = 32, 26.9%) than comparison group (*n* = 33, 16.9%) at 12 months (RR = 1.72, *p* = 0.016) Clinical outcome measures, behaviors measures: none Process measures: Increased identification in depression, (5.8 to 16.4%, 95%CI, 2.9–19.1) and AUD (0–13.8%, 95%CI, 0.6–24.9)
Petersen, et al. 2021, South Africa [44]	12 months	Patient-reported measures: Proportion of patients with at least 50% reduction in PHQ-9 scores at 6 months, no significant difference, (*N* = 256/456 vs *N* = 232/492), (55.9% vs 50.9%, RR-0.04, 95%CI, -0.19–0.11, *p* = 0.6) Clinical outcome measures, behaviors measures: none
Salisbury, et al. 2018, UK [45]	15 months	Clinical outcome measures: Number of deaths, illness burden, number of hospital admissions or outpatient attendances, no evident of difference; Quality of life, no difference in the primary outcome of quality of life (adjusted difference in mean EQ-5D-5L 0.00, 95%CI, –0.02–0.02; *p* = 0.93) Patient-reported measures, behaviors measures: none Process measures: Patient-centered care, all measures of patient-centered care showed benefits from the intervention after 15 months; Process of care, a significant difference in the Continuity of Care Index; Quality of disease management and number of indicators of high-risk prescribing, no evident of a difference; Consultations, more nurse consultations and more primary care physician consultations over 15 months in the intervention group, no evidence of difference
Sharpe, et al. 2014, UK [46]	12 months	Clinical outcome measures: Less depression, anxiety, pain, and fatigue; Better functioning, overall health, quality of life (all *p* < 0.05) Treatment response (at least a 50% reduction in depression severity from baseline on the self-rated SCL-20); The primary outcome of treatment response was achieved by 62% (143/231) of participants allocated to depression care for people with cancer and 17% (40/231) of those allocated to usual care; The absolute difference in outcome between the treatment groups was 45% (95% CI 37–53%); The OR for the treatment response in the two groups was 8.5 (95% CI, 5.5–13.4, *p* < 0.0001) Patient-reported measures: better perceived quality of depression care at all timepoints (*p* < 0.05) behaviors measures, process measures: none
Towfighi, et al. 2021, USA [47]	12 months	Clinical outcome measures: Mean systolic BP, improved from 143 (17) mm Hg at baseline to 133 (20) mm Hg at 12 months in the intervention group and from 146 (19) mm Hg at baseline to 137 (22) mmHg at 12 months in the usual care group, with no differences in the change between groups; Serum CRP level: greater improvements in CRP level (difference in log CRP = − 0.4, 95%CI − 0.7- − 0.1, *P* = 0.003); LDL, HbA1c, BMI, no differences; Patient-reported measures: none Behaviors measures: reduced consumption in self-reported salt intake (difference, 15.4, 95% CI 4.4–26.0, *P* = 0.004); Antithrombotic adherence, physical activity level, diet, smoking status, no differences Process measures: none
Walker, et al. 2014, UK [53]	32 weeks	Clinical outcome measures: Self-rated depression improvement, anxiety, quality of life, role functioning, perceived quality of care, all improved in the treatment group; Patient-reported measures: Average depression severity, significantly lower in patients allocated to depression care for people with lung cancer (mean score on the SCL-20 1.24 (SD 0·64)) than in those allocated to usual care (mean score 1.61 (SD 0.58)), difference = –0·38 (95% CI –0.58- –0.18), difference = –0.62 (95% CI –0.94- –0.29) Behaviors measures, process measures: none
Wolff, et al. 2021, USA [49]	12 months	Clinical outcome measures: Significantly lower HbA1c (β = − 0.14, *P* = 0.02) at 12 months; Significantly lower PHQ-9 scores (β = − 0.39, *P* = 0.03) at 12 months; BP, quality of life, no significant difference Patient-reported measures, behaviors change, process measures: none
Wood, et al. 2008, USA [50]	1 year	Clinical outcome measures: BP, target of less than 140/90 mm Hg was attained by both coronary (615 (65%) vs 547 (55%), difference = 10.4%, 95%CI 0.6–20.2, *p* = 0.04) and high-risk (586 (58%) vs 407 (41%), difference = 16.9%, 95%CI 2.0–31.8, *p* = 0.03) patient-reported measures: Total cholesterol of less than 5 mmol/L did not differ between groups; in high-risk patients the difference in change from baseline to 1 year was 12.7% (95%CI 2.4- 23.0, *p* = 0.02) in favor of INT Behaviors measures: no difference in those who did not smoke 1 year afterwards (difference = 10.4%, 95% CI − 0.3–21.2, *p* = 0.06); reduced consumption of saturated fat (196 (55%) vs 168 (40%), 17.3%, 6.4 to 28.2, *p* = 0.009); increased consumption of fruit and vegetables (680 (72%) vs 349 (35%), 37.3%, 18.1 to 56.5, *p* = 0.004), and oily fish (156 (17%) vs 81 (8%), 8.9%, 0.3 to 17.5, *p* = 0.04) at 1 year; High-risk individuals and partners showed changes only for fruit and vegetables (*p* = 0.005) Process measures: higher prescriptions for statins in the hospitals (difference = 6.0%, 95%CI − 0.5–11.5, *p* = 0.04); more frequent prescribed for angiotensin-converting enzyme inhibitors (difference = 8.5%, 95%CI 1.8–15.2, *p* = 0.02) and statins (difference = 14.6%, 95%CI 2.5–26.7, *p* = 0.03)

Ten studies presented data on mental health outcomes (Table 2). All of the ten studies showed significant improvements in depression-related measurements. Four studies reported statistically significant improvements in depression severity [34, 46, 52, 53]. Aragonès et al. reported the response rate to antidepressant treatment was 18.9% higher than the control group (OR = 2.74, 95%CI = 1.12–6.67); Katon et al. found that the interventions group was more likely to have a 50% decrease in SCL-90 depression score (OR = 1.62 (95%CI = 0.98–2.67) at 6 months, and OR = 1.47 (95%CI = 0.90–2.39) at 12 months; Sharpe et.al. detected a 50% reduction on SCL-20 (OR = 8.5, 95%CI = 5.5–13.4, *P* < 0.001), and Walker et al. found a 0.62 decrease in SCL-20 score (95%CI = -0.94–0.29). Seven studies presented data on mean depressive scores or PHQ-9 scores, or SCL-20 depression scores. Only one study showed no significant difference in the reduction in PHQ-9 score [44]. Six studies showed significant improvements in the reduction of mean depressive scores or PHQ-9 scores or SCL-20 depression scores [34, 36, 40, 42, 43, 49]. Three studies showed significant improvements in anxiety [36, 46, 53]. One study showed a significant reduction of the generalized anxiety disorder scale (GAD-7) [36].

Three studies reported results of hospitalizations, mortality, and illness burden (Table 2). One study reported statistically significant lower hospitalization for COPD/asthma (OR = 0.91, 95%CI = 0.87–0.94) and diabetes or its complications (OR = 0.87, 95% CI = 0.83–0.92) [38]. Another two studies both reported no significant difference in the reduction in all-cause hospitalizations [37] or the number of hospital admissions/outpatient attendances or the number of deaths [45].

The three types of PCTs showed mixed results on clinical outcome measures (Table 3). Despite different measures reported, the three types of PCTs all reported important improvements in measures related to mental and psychological health. The differences between three types of PCTs, however, were not able to lead to a consistent result.

**Table 3:** Quality of care by types of different primary care teams (PCTs)

PCT types and Number of studies (%)	Outcome measures			Process measures
	**Clinical outcome measures**	**Patient-reported outcomes and experiences**	**Changes in patient behaviors**	
Upward PCT 7 (41.2%)	**Positive results:** - Lower severity in depression (SCL-20); improvements in anxiety, quality of life, role functioning [49]; - Lower PHQ-9 scores and HbA1c [52]; - Less depression, anxiety, pain, and fatigue; better functioning and quality of life [48]; - Lower in glycated hemoglobin levels, LDL cholesterol levels, systolic BP, SCL-20; better quality of life [44]; - Less severity of depression [40] **Negative results:** - No significant difference in BP, quality of life [52]; - No significant difference in reduction of PHQ-9 score, BP, BMI, waist circumference [46]; - No significant difference in HbA1c levels [40]	**Positive results:** - Improvements in self-rated depression, perceived quality of care [49]; - Better perceived quality of depression care, overall health [48]; - Greater satisfaction with care for diabetes, CAD or both and care for depression [44]; - Greater satisfaction with care, higher self-rated global improvement [40] **Negative results:** - Not reported	**Positive results:** - Increased monitoring of BP and glucose **Negative results:** - No significant difference in smoking [46]; - No significant difference in medication adherence [41]	**Positive results:** - Increased anti-depressants, insulin and antihypertensive medications initiation and adjustment [41]; - Increased insulin adjustments, antihypertensive medications and antidepressants [44]; - Greater improvements in adequacy of dosage of antidepressant medication treatment [40] **Negative results:** - No significant difference in receipt of counselling, receipt of antidepressant treatment [46]
Downward PCT 2 (11.8%)	**Positive results:** - Greater improvements in serum CRP level [47] - Greater improvements in PHQ-9 [43] **Negative results:** - No significant difference in changes of systolic BP^43;^ - No significant difference in LDL, HbA1c, BMI [47];	Not reported	**Positive results:** - Greater improvements in self-reported salt intake [47]; **Negative results:** - No significant difference in antithrombotic adherence, physical activity level, diet, smoking status [47]	**Positive results:** - Increased identification of depression and alcohol use disorder [43] **Negative results:** Not reported
Traditional PCT 8 (47.1%)	**Positive results:** - Improvements in BP control and total cholesterol control in high-risk patients [50]; - Improvements in depression [42]; - Improvements in depression, anxiety [36]; - Improved mental components [37]; - Lower severity of depression, higher response rate to antidepressant treatment and remission rate [34]; - Improved quality of life [37] **Negative results:** - No significant difference in quality of life, number of deaths or illness burden [45]; - No significant difference in all-cause hospitalizations [37]; - No significant difference in quality of life, self-efficacy, or disability [36]; - No significant difference in pain severity or pain response rate [34]	**Positive results:** - Improvements in patient-centeredness [45]; - Improvements in general health [37]; - Improved satisfaction with care, more patient-centered [36] **Negative results:** - No significant difference in illness perceptions or social support [36]	**Positive results:** - Reduced consumption of saturated fat, increased consumption of fruit, vegetables, and oily fish [50]; - Increased exercise, increased visits to mental health workers [42]; - Lower hospitalizations for ambulatory care sensitive conditions [38]; - Better in self-management [36] **Negative results:** - No significant difference in hospital admissions or outpatient attendances [45]; - No significant change in smoking [50]	**Positive results:** - More prescriptions of statins, angiotensin-converting enzyme inhibitors [50]; - Improvement in the Continuity of Care Index [45]; - Increased referrals to exercise program, mental health workers [42]; - Increased self-management plan formulation, assessment of smoking status, measuring BMI [35]; - More consultations [45] **Negative results:** - No significant difference in quality of disease management and high-risk prescribing [45]; - Decreased testing for LDL; no significant difference in testing for HbA1C, HgbA1C, LDL, and BP [35]

#### Patient-reported outcomes and experiences

Seven studies presented patient-reported health outcomes and experiences (Table 2). Four studies reported patients’ satisfaction with care, and all found significant greater satisfaction [34, 36, 39, 40], for example, Katon et al. found that patients receiving care from PCTs were significantly more satisfied with care after 6 months (OR = 2.01; 95% CI = 1.18–3.43) and 12 months (OR = 2.88, 95% CI = 1.67–4.97) [39]. For perceptions of improvement, four studies reported this measure and all found better results in the intervention group [34, 39, 46, 48], although one study found the effect “slight more favorable” because the average point scores lie between categories of “no change” and “a little better” (Mean = 3.52 vs 3.97, *P* = 0.011) [34]. Studies also found care was more patient-centered delivered by PCTs [36, 45], for example, the standardized difference of patient-centeredness measured by PACIC was 0.39.

Studies of downward PCTs did not present the patient-reported outcomes and experiences, which was subjected to risk of reported bias. The upward PCTs and downward PCTs both performed better in this aspect. Patients were found to have better perceived quality of care [46, 48], and greater satisfaction with care [39, 40]. The traditional PCTs seemed to enhance patient-centeredness better, with two studies reported improvements in this measure [36, 45].

#### Changes in patient behaviors

Nine studies reported outcomes on patient behaviors (Table 2). Four studies reported medication adherence, and only one study found significant improvement (OR = 2.18, 95%CI = 1.32–3.62) [39]. Seven studies provided results of patients’ lifestyle changes. The three studies that reported smoking status found no significant improvement [44, 47, 50]. Findings on exercises were mixed, Morgan et al. reported a 19% increase in exercise [42], while the other two studies did not find significant improvement [40, 47]. As for diet habits, one study found improvement in self-reported salt intake (Difference = 15.4, 95% CI = 4.4–26.0, *P* = 0.004) [47]. Another study found that the intervention group reduced saturated fat intake by 17.3% (95%CI = 6.4–28.2, *P* = 0.009) and increased intake of fruit and vegetables by 37.3% (95%CI = 18.1–56.5, *P* = 0.004) [50].

Traditional PCTs and downward PCTs had better performance than upward PCTs for patient behaviors. In the traditional PCTs and down PCTs, patients changed their diet habits and exercise. Wood et al. found patients reduced consumption of saturated fat and increased consumption of fruit, vegetables and oily fish, and Towfighi et al. also found patients reported they reduced their salt intake [47, 50].

#### Process measures

Five studies reported process measures (Table 2). Chen et al. reported that the change in the testing for HbA1c did not differ between the intervention and control group, but testing for LDL was significantly lower in the intervention group (difference = -5.8%, *P* = 0.001) [35]. For the intervention group, measurement of BMI (+ 85%, *P* < 0.001), assessing smoking status (+ 82.8%, *P* < 0.001), making self-management plan (+ 35.6%, *P* < 0.001) increased significantly compared with baseline. Lin et al. found that initiation and adjustment of medication increased significantly in the intervention group, and the RR was 6.2 (*P* < 0.001), 1.86 (*P* < 0.001), and 2.97 (*P* < 0.001) for antidepressants, antihypertensive drugs, and insulin, respectively. Salisbury reported consultations and continuity of care for process measure [45]. The continuity of care was significantly higher in the intervention group (Difference = 0.08, 95%CI = 0.02–0.13, *P* = 0.0045). Patients in the intervention group also had more consultations with primary care nurses (Difference = 1.37, 95%CI = 1.17–1.61, *P* < 0.001) and primary care physicians (Difference = 1.13, 95%CI = 1.02–1.25, *P* = 0.021), but not in hospital admissions (Deference = 1.04, 95%CI = 0.84–1.30, *P* = -0.71) and hospital outpatient attendances (Difference = 1.02, 95%CI = 0.92–1.14, *P* = 0.72). Katon et al. also found that patients in the intervention group were more likely to revive medication adjustment, including insulin (*P* = 0.006), antihypertensive agents (*P* < 0.001), and antidepressants (*P* < 0.001) [40]. Morgan et at. also detected more consultations to mental health workers in the intervention group (+ 17% vs -3%).

Two studies of upward PCTs reported medication adjustments, for example, insulin and antihypertensive medications [40, 41]. For traditional PCTs, Wood et al. found more prescriptions of statins, angiotensin-converting enzyme inhibitors were provided [50]. Studies of traditional PCTs had more process measures reported than the other two types of PCTs, and also found a great deal of changes in processes. Some of the changes, such as increased self-management plan formulation, showed a promising effect for patients with multimorbidity [35].

### Mechanisms of PCTs on quality of care

The discussions of the mechanisms of PCTs on quality of care in the seventeen studies were extracted and reported in Table S1 (supplementary file 3). The articles highlighted several common mechanisms by which PCTs achieved effective or ineffective results. First, support for team members and patients from leadership was fundamental and foremost for PCTs implementation and effectiveness [35]. Chen et al. considered that active participation and support from departmental leadership was critical in implementing and sustaining the Teamlet intervention. Second, changing the organization of care played an important role in increasing coordination of care, for example, integrating mental care [36, 49] or cancer care [48] with primary care. Integrating mental care for patients with chronic conditions comorbid with depression reduced depressive symptoms in patients with chronic conditions, but how to integrate mental and physical healthcare in patients with broader multimorbidity was not certain [36]. Third, timely follow-up and medication adjustment were important to enhance medication adherence and achieve treatment goals [40, 41]. Adjusting medication portfolio timely could guarantee a higher chance of improvements in clinical goals and also serve as a reminder for patients’ adherence. Fourth, strong support for patients’ self-management provided by PCTs was reported to contribute strongly to improving quality of care [40–42, 47]. Nurses educated patients about essential skills and knowledge in managing their conditions and changing their lifestyle [39].

## Discussion

As the chronic disease burden increase and more patients with multimorbidity, primary care faces more challenges in balancing the increasing disease burden and assuring quality of care. PCTs have been examined in developed countries to cope with this transition. This review examined the characteristics of PCTs, the impacts of PCTs on quality of care, and the mechanisms by which PCTs influence quality of care in primary care settings among patients with multimorbidity. First, PCTs designed for people with multimorbidity can be broadly categorized into three types: 1) upward collaborative PCTs (primary care workers and specialists), 2) downward PCTs (primary care workers and lay health workers), and 3) traditional PCTs (primary care physicians and care managers). Second, as for the impact of PCTs on quality of care among people with multimorbidity, PCTs have improved patients’ mental and psychological health outcomes, improved patients’ perceptions towards care, changed the process of care, and showed mixed effects on clinical outcome measures. Third, as for the mechanisms of PCTs on quality of are among people with multimorbidity, 1) support for team members and patients from leadership, 2) re-organization of care, 3) timely follow-up and medication adjustment, and 4) strong support for patients’ self-management provided by PCTs contributed to the effectiveness of PCTs.

A team environment could provide support for the health workers involved as well as enable more efficient sharing of information [54]. In the included studies, different terms were used to describe a range of interventions delivered by several health workers working as a team to provide health services, such as collaborative care, integrated care, group practice, etc. Despite varying content or intensity of interventions, the core was the manner of working as a team.

The three models of PCTs faced different challenges in implementation and achieving desired outcomes. For the upward collaborative PCTs, medical specialists from upper-level hospitals were engaged. The most important role for specialists was to train primary health workers, especially in less developed regions where primary health workers did not have enough capacity to manage patients with complex needs. For ensuring the quality of managing patients with multimorbidity, appropriate training and timely guidance from specialists are especially helpful for GPs and nurses. However, over-reliance on specialists could undermine the roles of GPs in the team and underscore perceptions that the wellbeing of patients is not the team’s responsibility. Specialists are prone to become the team’s center because of the imbalanced power [55], which is inappropriate when they are not engaged in most care delivery or patient contacts in PHC settings.

For the downward collaborative PCTs, the non-clinical procedures in a patient journey were more taken as the health system's responsibility than the other two types of PCTs, which was carried out by the lay health workers. There were several advantages involving lay workers from the community into the team. First, they live near to patients and can have more interactions with patients and thus increase the continuity of care, and also reinforce the effect of self-management [47]. Second, they have more flexible time to help patients with non-medical matters like transportation to hospitals. However, patients may not trust them because they are not medical professionals, which may decrease the team’s effectiveness. In the two studies of the downward collaborative PCTs, the community health workers also provided counseling services, medical education to patients and activated emergency medical services when needed [43, 47], which means that they had to be trained and upskilled about the knowledge for specific disease [56]. Their qualification and scope of providing the services are also of concern. The communication and administration costs also increase as the team becomes larger.

For traditional PCTs, GPs and care managers have already worked collaboratively under the same roof of a clinic. How to mobilize the health workers in a more effective way to improve the quality of care is the renewed interest. The team was used to be led by GP [57], but new PCTs empowered nurses/medical assistants to lead the team. Traditional PCTs used the existing workforce without system-level changes, making the model more generalizable for other places with funding constraints. The smaller scale of the PCTs and shorter distance among team members decrease the communication and administration cost. Besides, a clear division of labor should be determined so that full potential can be brought out of the staff [58]. However, the extra responsibilities, such as giving an overall assessment of patients, helping patients to develop goals, were mostly assigned to nurses or medical assistants. Nurses or medical assistants’ workload increased greatly, especially for patients with multimorbidity who require more attention to manage.

PCTs achieve effectiveness by several mechanisms. First, support for team members and patients from leadership plays an important role in enhancing team members’ confidence and enthusiasm [35, 45]. Second, re-organization of care ensure, including enhancing integration of care [36, 38, 40, 49, 50, 52], accessibility of care comprehensiveness of care and continuity of care [35, 38, 46, 50, 53]. Third, timely follow-up and medication adjustment increase the intensiveness of care [46, 53, 59, 59]. Lastly, PCTs provide strong support for patients’ self-management, which also contribute to the effectiveness of PCTs.

Taking three types of PCTs together, we can see that some extra responsibilities are added to the health system: first, self-management is no longer patients’ own business, but helped and guided by the health system; second, non-clinical procedures in patient journeys are also taken care of by the health system; patients’ wellbeing especially mental health instead of individual disease are emphasized more in improving health. PCTs provided an opportunity to rearrange those extra workloads. Routine patient management was transferred to care managers or coordinators that included nurses, medical assistants, community health workers or lay counselors, etc. Besides non-clinical contact with patients, nurses in some models also took over some therapeutic work from physicians, such as screening and monitoring depression. The efficient implementation of PCTs depends largely on nurses’ capability. They decide appropriate and feasible self-management measures for patients with multimorbidity, and also serve as the link between patients and the health system.

When working as a team, health workers have opportunities to supervise and correct each other in daily clinical practice, which may contribute to the improved process of care. The PCTs also bring more interaction between patients and the health workers. Those increased face-to-care communications can make patients learn more about their conditions and also more about their PCTs, and help them to order the chaos in multiple morbidities [60]. The influence of changes in prescribing behaviors including medication adjustments on quality of care for people with multimorbidity needs to be studied in the future. Timely adjusting medication can have positive effects on patients, but whether increased adjustments will lead to over reliance on medications and overlooking self-management is unclear.

Inadequate collaboration within teams is a primary cause for studies that showed unsatisfactory improvements. PCTs provide an opportunity to learn from other team members and offer holistic, continuous, and comprehensive care for patients with complex needs [13]. However, whether care is actually provided by a team remain questioned. A previous review that examined collaborative care among cancer patients comorbid with depression found that collaborative care is promoted but not achieved. The current models were hospital-based and reliant on medication as the primary treatment [61]. In this review, studies also reported the similar problem. Only 14.5% of participants received the intended full components of interventions [47], and only 49% of intervention participants received full intervention package [45]. There may be defects in the studies’ process, but it also highlights implementation problems in real world. From design to practice, PCTs may take more time and efforts than expected.

The effective implementation of PCTs demands support from external environments, otherwise if would lead to ineffective results. Most studies identified in this review were RCT, but the incentive mechanisms for the team were not well designed in the interventions. This is of concern for a wider implementation, especially when involving health workers outside clinics. Besides, the payment mechanism should also be updated to fit in this new working model. The introduction of computerized tools in PCTs is also noteworthy. Working with multiple people requires communication and coordination. Information acquired from the previous health worker about patients’ needs to be passed quickly and efficiently to the next team member. Some interventions introduced such tools to facilitate the work process by integrating a computerized decision support system and clinical electronic medical record system [34]. It helped all team members to share the information at any time, and served as a complement for routine meetings. This is consistent with the development of care for multimorbidity. Data-informed care has been propelled for managing patient with multimorbidity at both the individual and population level [62]. Besides, governance and management of PCTs are also important to sustain PCTs and allow for continuity of care. The organization of the PHC system varies significantly in different contexts, so there is no one PCTs fit for all regions or health systems. When interpreting the effectiveness of PCTs, it’s important to remember the contexts of that PCTs, especially when implementing it elsewhere.

### Limitations

This systematic review was subject to several limitations. First, due to the complexity of interventions, we could not link a specific intervention with the changes in quality of care. Second, the strict inclusion and exclusion criteria of patients and heterogeneity in specific diseases of multimorbidity in the literature limited the generalizability of the results, especially given the complex profile of patients involved in the real world. Third, the quality of training provided as part of the interventions was unable to be assessed in this review. Our analysis of the interventions was limited by the published descriptions of interventions. Although we also searched for published protocols and other information when the descriptions were not detailed, it might still encounter publication bias.

## Conclusion

This review has evaluated the PCTs and their impact on quality of care for patients with multimorbidity and suggested major gaps in the evidence that needs to be filled. On the basis of our results, we recommend that future research should further explore the impact of PCTs on clinical outcome measures for patients with multimorbidity. Moreover, the providers’ and patients’ attitudes and acceptability of the PCTs should be examined to inform policy makers of the implementation difficulties. To move from research to practice, policy makers should design incentives for the teams and their members, clarify the job responsibilities for different roles without compromising the quality of care or over-reliance on specialists, and embrace technology to increase efficiency of care delivery.

## Supplementary Information

**Additional file 1.** Search strategies.

**Additional file 2.** Cochrane criteria for risk of bias and outcomes & MMAT check lists.

**Additional file 3:** **Table S1. **Mechanisms for effective results and ineffective results for primary care teams (PCT).

## Data Availability

All data generated during this research are incorporated in the article and its online supplementary material.

## Abbreviations

PCTs: Primary care teams
PHC: Primary health care
RCT: Randomized controlled trials
GP: General practitioners
