# Supplementary material for: Primary care team and its association with quality of care for people with multimorbidity: a systematic review
Source: BMC Prim Care. 2023 Jan 19;24:20. doi: 10.1186/s12875-023-01968-z (PMC9850572; doi:10.1186/s12875-023-01968-z)
Supplement: Supplementary file 1 — Additional file 1. Search strategies. [file 12875_2023_1968_MOESM1_ESM.pdf]

Primary Care Team and its Association with Quality of Care for People with Multimorbidity: A Systematic Review

Supplementary file 1: Search Strategies

Search date: November 2nd, 2021

1. Database: Ovid Medline ® <January 2000 to October week 4 2021>

Concepts	Research strategy keywords	No.	Results
Population	chronic disease/	1	271807
	(chronic adj (disease? or illness$ or care)).tw.	2	88885
	long-term care/	3	27161
	((longterm or long-term) adj care).tw.	4	22318
	comorbidity/	5	118784
	(comorbid$ or co-morbid$).tw.	6	212168
	((co-existing or coexisting) adj (illness$ or disease$)).tw.	7	1262
	(multimorbid$ or multi-morbid$).tw.	8	6945
	or/1-8	9	647770
	exp diabetes mellitus/	10	459387
	exp hypertension/	11	300459
	exp heart diseases/	12	1193087
	exp cerebrovascular disorders/	13	394392
	exp asthma/	14	134435
	exp pulmonary disease/	15	1045404
	exp thyroid diseases/	16	154039
	exp hyperlipidemia/	17	68190
	exp arthritis rheumatoid/	18	118513
	exp mental disorders/	19	1329393
	exp substance-related disorders/	20	293711
	exp epilepsy/	21	117907
	exp hiv infections/	22	298173

	exp neoplasms/	23	3563695
	exp kidney diseases/	24	539258
	exp liver/	25	456583
	exp osteoporosis/	26	58938
	exp tuberculosis/	27	198686
	or/10-27	28	8977198
	9 and 28	29	319257
Setting	exp primary health care/	30	176458
	exp physicians, family/	31	16800
	(family medicine).tw.	32	11386
	exp community health care/	33	318930
	(primary adj2 care).tw.	34	147823
	((general or family) adj pract$).tw.	35	93916
	(community health adj (services or care)).tw.	36	2071
	(general practitioners).tw.	37	36618
	or/30-37	38	634171
	29 and 38	39	19104
intervention	exp patient care team/	40	71274
	exp group practice/	41	23587
	((primary or community or rural) adj3 (team* or model or network)).tw.	42	21277
	((interdisciplinary or inter-disciplinary or multiprofessional or multi-professional) adj5 (team* or model)).tw.	43	8346
	(team-based).tw.	44	4654
	(health* adj5 (team or model)).tw.	45	41655
	(general practitioner$ adj5 (team* or model)) .tw.	46	471
	(integrat* adj5 (team* or model)) .tw.	47	23809
	(health adj5 teamwork) .tw.	48	539
	(health adj5 network) .tw.	49	9030

	((general practitioner$) or (health* worker$) or physician or doctor$ or (health professional$)) and (team or model or network) .tw.	50	56154
	(social worker or pharmacist or dentist or psychiatrist) AND (team* or model or network)	51	4791
	(integrat$ and (team or model or network)) and (health$ or care).tw.	52	42596
	or/40-52	53	253184
	39 and 53	54	2434
Restrictions	54 not (comment or editorial or letter).pt.	55	2400
	55 not (meta-analysis).pt.	56	2387
	limit 56 to yr= “2000 -Current”	57	2031
	limit 57 to english language	58	1851

Search date: 2021 年 11 月 2 日星期二

2. Database: PubMed <January 2000 to October week 4 2021> 845

Concepts	Research strategy keywords	No.	Results
Population	Chronic Disease [TIAB] OR Chronic Illness [TIAB] OR Chronic Care [TIAB]	#1	56,000
	Long-Term Care [MH] OR longterm care [TIAB]	#2	27,243
	Comorbidity [MH] OR Co-morbidity [TIAB]	#3	127,400
	Multimorbidity [MH] OR Multi-morbidity [TIAB]	#4	2,445
	Multiple Chronic Conditions [MH]	#5	581
	Co-existing Illness [TIAB] OR Coexisting Illness [TIAB] OR Co-existing Disease [TIAB] OR Coexisting Disease [TIAB]	#6	490
	#1 OR #2 OR #3 OR #4 OR #5 OR #6	#7	209,086
	Diabetes Mellitus [MH] OR Hypertension [MH] OR Heart Diseases [MH] OR Cerebrovascular Disorders [MH] OR Asthma [MH] OR Lung Diseases [MH] OR Thyroid Diseases [MH] OR Hyperlipidemias [MH] OR Arthritis, Rheumatoid [MH] OR Mental Disorders [MH] OR Substance-Related Disorders [MH] OR Epilepsy [MH] OR HIV Infections [MH] OR Neoplasms [MH] OR Kidney	#8	8,915,268

	Diseases [MH] OR Liver Diseases [MH] OR Tuberculosis [MH] OR Osteoporosis [MH]		
	#7 AND #8	#9	125,596
Setting	Primary Health Care [MH] OR Primary Care [TIAB] OR [Primary healthcare [TIAB]	#10	254,515
	Physicians, Family [MH]	#11	16,794
	General Practice [MH] OR Family Practice [MH] OR Family Medicine [TIAB]	#12	82,672
	Community Health Services [MH] OR Community Care [TIAB]	#13	321,774
	General Practitioners [MH]	#14	9,159
	#10 OR #11 OR #12 OR#13 OR #14	#15	2,866,505
	#15 AND #9	#16	23,294
Intervention	patient care team [MH]	#17	71,287
	primary team [TIAB] OR primary health team [TIAB] OR primary healthcare team [TIAB] OR primary health model [TIAB] OR primary healthcare model [TIAB] OR primary care team [TIAB] OR primary care model [TIAB]	#18	1,265
	interdisciplinary team [TIAB] OR interdisciplinary model [TIAB] OR inter-disciplinary team [TIAB] OR inter-disciplinary model [TIAB] OR multiprofessional team [TIAB] OR multi-professional model [TIAB]	#19	4,317
	team-based [TIAB] AND (health [TIAB] OR care [TIAB] OR healthcare [TIAB])	#20	3,572
	health team [TIAB] OR healthcare team [TIAB] OR health care team [TIAB] AND (primary [TIAB] OR community [TIAB] OR rural [TIAB])	#21	2,565
	(general practitioner [TIAB] OR health worker [TIAB] OR healthcare worker [TIAB] OR physician [TIAB]OR doctor [TIAB] OR health professional [TIAB]) AND (team [TIAB] OR model [TIAB] OR network [TIAB])	#22	33,249
	(social worker [TIAB] OR pharmacist [TIAB] OR dentist [TIAB] OR [TIAB] psychiatrist [TIAB]) AND (team [TIAB] OR model [TIAB] OR network [TIAB])	#23	71
	(integrated [TIAB] OR integration [TIAB]) AND (team [TIAB] OR model [TIAB] OR network [TIAB]) AND (health [TIAB] OR care [TIAB] OR healthcare [TIAB])	#24	27,269
	Group Practice [MH]	#25	23,587

	teamwork [TIAB] AND (health [TIAB] OR care [TIAB] OR healthcare [TIAB])	#26	7,407
	#17 OR #18 OR #19 OR #20 OR #21 OR #22 OR #23 OR #24 OR #25 OR #26	#27	158,954
	#16 AND #27	#28	1,036
Restrictions	#28 NOT (Letter [PT] OR news [PT] OR comment [PT] OR editorial [PT] OR bibliography [PT])	#29	1,021
	#29 NOT Meta-Analysis [PT]	#30	1,010
	#30 FILTERS: English, from 2000/1/1-2021/10/29	#31	845

Search date: November 3rd, 2021

3. Database: EMBASE <January 2000 to October week 4 2021>

Concepts	Research strategy keywords	No.	Results
Population	'chronic disease'/exp	#1	211763
	('chronic disease?' OR 'chronic illness' OR 'chronic care'):ab,ti	#2	73486
	((longterm OR 'long-term') NEAR/2 care):ab,ti	#3	32859
	comorbidity/exp	#4	316827
	(comorbid* OR co-morbid*):ab,ti	#5	377,975
	((co-existing OR coexisting) NEAR/2 (illness* OR disease*)):ab,ti	#6	3871
	(multimorbid* OR multi-morbid*):ab,ti	#7	9331
	#1 OR #2 OR #3 OR #4 OR #5 OR #6 OR #7	#8	764099
	'diabetes mellitus'/exp	#9	1102673
	hypertension/exp	#10	833604
	'heart diseases'/exp	#11	2150600
	'cerebrovascular disorders'/exp	#12	805860
	asthma/exp	#13	288992
	'pulmonary disease'/exp	#14	1890682
	'thyroid diseases'/exp	#15	273681
	hyperlipidemia/exp	#16	175480

	‘arthritis rheumatoid’/exp	#17	232306
	‘mental disorders’/exp	#18	2512536
	‘substance-related disorders’/exp	#19	269,521
	epilepsy/exp	#20	268,157
	‘hiv infections’/exp	#21	404,688
	neoplasms/exp	#22	5,372,463
	‘kidney diseases’/exp	#23	1,110,543
	‘liver diseases’/exp	#24	1,142,597
	osteoporosis/exp	#25	141,386
	tuberculosis/exp	#26	279,117
	#9 OR #10 OR #11 OR #12 OR #13 OR #14 OR #15 OR #16 OR #17 OR #18 OR #19 OR #20 OR #21 OR #22 OR #23 OR #24 OR #25 OR #26	#27	14093615
settings	‘primary health care’/exp	#28	185302
	(primary NEAR/2 care):ab,ti	#29	198,307
	‘family physicians’/exp	#30	107,462
	‘family medicine’/exp	#31	11,922
	‘family practice’/exp	#32	87,470
	((general OR family) NEAR/2 pract*):ab,ti	#33	135,845
	‘community health’/exp	#34	212,375
	(‘community health’ AND (service* OR care)):ab,ti	#35	17,876
	(general pract*):ab,ti	#36	232,571
	#28 OR #29 OR #30 OR #31 OR #32 OR #33 OR #34 OR #35 OR #36	#37	749802
Interventions	‘patient care team’/exp	#38	907955
	(primary NEAR/3 (team* OR model OR network)):ab,ti	#39	17722
	((interdisciplinary OR inter-disciplinary OR multiprofessional or multi-professional) NEAR/5 (team* OR model OR network)):ab,ti	#40	14046
	team-based:ab,ti	#41	6859
	(health* NEAR/5 (team* or model OR network)):ab,ti	#42	81272

	(‘general practitioner*’ NEAR/5 (team* OR model OR network)):ab,ti	#43	1127
	(integrat* NEAR/5 (team* OR model OR network)):ab,ti	#44	40700
	(‘group practice’):ab,ti	#45	4044
	(health NEAR/5 teamwork):ab,ti	#46	642
	(health NEAR/5 network):ab,ti	#47	12896
	((‘general practitioner*’) OR (‘health* worker*’) OR physician or doctor* OR (‘health professional*’)) AND (team* OR model OR network):ab,ti	#48	142177
	(‘social worker’ OR pharmacist OR dentist OR psychiatrist) AND (team* OR model OR network):ab,ti	#49	19701
	#38 OR #39 OR #40 OR #41 OR #42 OR #43 OR #44 OR #45 OR #46 OR #47 OR #47 OR #48 OR #49	#50	1153177
	(#8 AND #27) AND #37 AND #50	#51	79057905
restrictions	#51 NOT (‘interview’/de OR ‘animal cell’/de OR ‘animal experiment’/de OR ‘animal model’/de OR ‘animal tissue’/de)	#52	7528
	#52 NOT (‘meta analysis’/de OR ‘systematic review’/de)	#53	7312
	#53 AND [01-01-2000]/sd NOT [29-10-2021]/sd	#54	7121
	#54 AND [english]/lim	#55	6852

Search date: November 3rd, 2021

4. Database: ProQuest <January 2000 to October week 4 2021>

Concepts	Research strategy keywords	No.	Results
population	MESH(chronic disease)	S1	402941
	TI,AB(chronic NEAR (disease? OR illness$ OR care))	S2	661478
	MESH(long-term care)	S3	30586
	TI,AB((longterm OR long-term) NEAR care)	S4	116044
	MESH(comorbidity)	S5	133042
	TI,AB(comorbid$ OR co-morbid$)	S6	96281
	TI,AB((co-existing OR coexisting) NEAR (illness* OR disease*))	S7	5801
	TI,AB(multimorbid* OR multi-morbid*)	S8	12070
	S1 OR S2 OR S3 OR S4 OR S5 OR S6 OR S7 OR S8	S9	1279197

	MESH(diabetes mellitus)	S10	406,953
	MESH(hypertension)	S11	352,439
	MESH(heart diseases)	S12	245,157
	MESH(cerebrovascular disorders)	S13	53,107
	MESH(asthma)	S14	144,982
	MESH(pulmonary disease)	S15	138,975
	MESH(thyroid diseases)	S16	36,854
	MESH(hyperlipidemia)	S17	30513
	MESH(arthritis rheumatoid)	S18	110,553
	MESH(mental disorders)	S19	233,350
	MESH(substance-related disorders)	S20	108,470
	MESH(epilepsy)	S21	119,816
	MESH(hiv infections)	S22	234,406
	MESH(neoplasms)	S23	3,168,666
	MESH(kidney diseases)	S24	192,817
	MESH(liver diseases)	S25	196,414
	MESH(osteoporosis)	S26	65,780
	MESH(tuberculosis)	S27	226395
	S10 OR S11 OR S12 OR S13 OR S14 OR S15 OR S16 OR S17 OR S18 OR S19 OR S20 OR S21 OR S22 OR S23 OR S24 OR S25 OR S26 OR S27	S28	5687491
	S9 AND S28	S29	306364
settings	MESH(primary health care)	S30	97,859
	MESH(physicians, family)	S31	39104
	TI,AB(family medicine)	S32	82,417
	MESH(community health care)	S33	52999
	TI,AB(primary NEAR care)	S34	330371
	TI,AB((general OR family) NEAR practice)	S35	141974
	TI,AB(community NEAR health NEAR (service* OR care))	S36	63,079
	TI,AB(general practitioner*)	S37	126862
	S30 OR S31 OR S32 OR S33 OR S34 OR S35 OR S36 OR S37	S38	742555
	S29 AND S38	S39	12389
Intervention	MESH(patient care team)	S40	74096

	MESH(group practice)	S41	31,109
	TI,AB((primary OR community OR rural) NEAR/3 (team* OR model OR network))	S42	169,702
	TI,AB((interdisciplinary OR inter-disciplinary OR multiprofessional OR multi-professional) NEAR/5 (team* OR model))	S43	22,892
	TI,AB(team-based)	S44	16378
	TI,AB(health* NEAR/5 (team OR model))	S45	186298
	TI,AB(general practitioner* NEAR/5 (team* OR model))	S46	1922
	TI,AB(integrat* NEAR/5 (team* OR model))	S47	198730
	TI,AB(health NEAR/5 teamwork)	S48	1450
	TI,AB(health NEAR/5 network)	S49	66019
	TI,AB((general practitioner$) OR (health* worker$) OR physician OR doctor$ OR (health professional$)) AND (team OR model OR network)	S50	726477
	TI,AB(social worker OR pharmacist OR dentist OR psychiatrist) AND (team* OR model OR network)	S51	153647
	TI,AB(integrat* AND (team OR model OR network)) AND (health$ OR care)	S52	259870
	S40 OR S41 OR S42 OR S43 OR S44 OR S45 OR S46 OR S47 OR S48 OR S49 OR S50 OR S51 OR S52	S53	1615668
	S39 AND S53	S54	2964
Restrictions	limit S54 to yr “20000101-20211029”	S55	2677
	S55 NOT STYPE(comment OR editorial OR letter)	S56	2145
	limit S56 to english language	S57	2049
	S57 NOT STYPE(interviews as topic)	S58	1975
