# Supplementary material for: Primary care team and its association with quality of care for people with multimorbidity: a systematic review
Source: BMC Prim Care. 2023 Jan 19;24:20. doi: 10.1186/s12875-023-01968-z (PMC9850572; doi:10.1186/s12875-023-01968-z)
Supplement: Supplementary file 2 — Additional file 2. Cochrane criteria for risk of bias and outcomes & MMAT check lists. [file 12875_2023_1968_MOESM2_ESM.pdf]

Primary Care Team and its Association with Quality of Care for People with Multimorbidity: A Systematic Review

Supplementary file 2: Cochrane Criteria for Risk of Bias and Outcomes & MMAT check lists

Reference	Adequate sequence generation	Adequate allocation concealment	Blinding addressed (participants, personnel, assessors)	Sample Size considerations	Incomplete outcome data addressed (Attrition)	Screening Criteria	Primary Outcome Measure	Analysis	Results
Aragonès, 2019, Spain	Computer-generated random assignment	Yes	Assessors	330; α= 0.05, power=80%, and a 15% dropout rate, expected intracluster correlation coefficient (ICC) = 0.01	At 12 months, 274 (83%) completed assessment.	Positive for moderate or severe pain (BPI) and major depression (PHQ-9) by phone	Severity of depression symptom: SCL-20	ITT	OR: −0.23 (95% CI=−0.42 to −0.04, P= 0.016)
Chen, 2010, USA	-	-	-	-	-	-	-	-	-
Coventry, 2015, UK	Minimisation of practices for multiple deprivation and list size	Yes	Assessors	360; 79% power to detect an effect of 0.4, α = 0.05, ICC =0.06, attrition=20%	At 4 months, 350 (90%) participants completed assessment.	Scored ≥ 10 on the PHQ-9 at the face-to-face visit	SCL-D13	ITT	OR: 0.23 (95% CI= −0.41 to −0.05, P=0.01)
Freund, 2016, Germany	Computer-generated random assignment	Yes	Assessors	2210; 80% power at the 5% significance level to detect a between group difference of 0.14,	At 12 months, 1875 (90%) completed assessment	Not listed	All-cause hospitalizations	ITT	12-month RR: 1.01 (95% =0.87–1.18)

				attrition=15%, design effect= 1.16, intracluster correlation of 0.01					24-month RR: 0.98 (95% CI, 0.85 to 1.12)
Jan, 2021, China	-	-	-	-	-	-	-	-	-
Katon, 2004, USA	Computerized algorithm	No	Assessors	300; attrition=15%, 80% power to detect as significant a 0.23 (SD, 0.7) difference in SCL-90;<br>324; attrition=15%, 80% power to detect as significant a 0.5 (SD, 1.65%)	At 12 months, 146 (88.5%) intervention group and 142 (86.1%) control group completed assessment	PHQ-9=10 or greater on the in the initial screening and persistent symptoms, SCL-90>1.1 at a second telephone screen 2 weeks later.	SCL-90	ITT	OR: 1.89 (95% CI=1.18-3.02)
Katon, 2010, USA	Permuted-block design	No	Assessors	145/group; 15% attrition, 80% power to detect a difference of 15% difference in patients achieving disease control on all three measures, $\alpha<0.05$	At 12-month, 83% completed all assessment.	PHQ-2 by mail or telephone and PHQ-9 by telephone, PHQ-2≥3 or PHQ-9≥10	SCL-20, glycated haemoglobin level, systolic blood pressure, and LDL cholesterol level	ITT	Difference: Glycated haemoglobin level: 0.58%; LDL: 6.9 mg/deciliter (0.2 mmol/liter); Systolic BP: 5.1 mm Hg; SCL-20: 0.40 (P<0.001)

Lin, 2012, USA	Permuted block design	No	Assessors	145/group; 15% attrition, 80% power to detect a difference of 15% difference in patients achieving disease control on all three measures, α<0.05	At 12-month, 181 (84.6%) had complete pharmacy data	PHQ-2 by mail or telephone and PHQ-9 by telephone, PHQ-2≥3 or PHQ-9≥10	Self-Monitoring and Medication Adherence	ITT	RR=6.20; P<0.001 for antidepressants; RR= 2.97; P <0.001 for insulin; RR=1.86, P<0.001 for antihypertensive medications
Morgan, 2013, Australia	Not listed	No	No	450; 80% power, α<0.05, an intracluster correlation of 0.04, a 50% dropout	14% (n=28) of patients withdrew, 4% leaving after 6 months, 5% after 9 months and 5% after the full year.	PHQ-9>5 by email	PHQ-9	ITT	Difference: decreased by 5.7±1.3 vs 4.3±1.2 in control (P=0.012)
Petersen, 2019, South Africa	-	-	-	-	-	-	-	-	-
Petersen, 2021, South Africa	Database software algorithm	No	Statistician	1000; intra-clinic correlation coefficient of 0.04 for a 50% improvement in PHQ-9,	56 (90%) and 492 (91%) at 6 months; 441 (88%) and 484	Positive on PHQ-9	PHQ-9	ITT	RD= -0.04 (95% CI = -0.19-0.11, P= 0.6)

				α<0.05, a 20% attrition, at least 80% power	(90%) at 12 months				
Salisbury, 2018, UK	Minimised by practice deprivation and list size	Yes	Assessors, personnel	32 practices and 1382 patients; 90% power (with a two-sided α of 0·05) to detect a difference of 0·274 SDs in the EQ-5D-5L; Assuming 108 eligible patients per practice, 40% agreeing to participate, 80% retention, and an ICC 0·03	1361 (88%) participants at 15 months (n=670 in control and n=691 in intervention)	With at least three types of chronic conditions	EQ-5D-5L	ITT	OR: 0·00, (95% CI 0·02 to 0·02; p=0·93)
Sharpe, 2014, UK	Database software algorithm	Yes	Assessors, statistician, personnel	500; 90% power, α<0.05, to detect a difference of at least 0.15 (15%)	At 24 weeks, 462 (92%) of the 500 participants (462 [96%] of 483 patients who were still alive)	Diagnosed with major depression by Structured Clinical Interview for DSM-IV face-to-face	SCL-20	ITT	OR: 8·5 (95% CI 5·5–13·4), p<0·0001
Towfighi, 2021, USA	Computer-assisted stratified randomization	No	Personnel	261; ICC= 0.0085, attrition=30%, power=80%, autocorrelation=0.2	412 (84.6%) completed the 12-month assessment	Systolic BP 130 or greater or 120 to 130 in individuals with a history of hypertension or	Systolic BP	ITT	Difference: −3.3 [95% CI=−14.9 to 8.8 mm Hg; *P* = 0.57

						using antihypertensive medications			
Walker, 2014, UK	Database software algorithm, with minimization of age, sex, and lung cancer type	Yes	Statisticians and personnel	150; 90% power at the 5% significance level to detect a standardised mean difference of 0·53 and 80% power to detect a standardised mean difference of 0·46	131 (92%) of 142 participants provided outcome data (59 [86%] in the intervention and 72 [97%] in the control)	Identified as having probable major depression Structured Clinical Interview for DSM-IV at face-to-face interview	SCL-20	ITT	Difference: -0·62 (95% CI –0·94 to –0·29)
Wolff, 2021, USA	-	-	-	-	-	-	-	-	-
Wood, 2008, USA	Not listed	No	No	400;80% power, α<0.05, cluster coefficient for smoking was 0·200, bodyweight 0·011, systolic blood pressure 0·030, and total cholesterol concentration 0·062	Not listed	SCORE ≥5% during 10 years, either now or when projected to age 60 years	Family-based lifestyle change at 1-year	ITT	Difference: 10.4%, (95% CI −0·3 to 21·2, p=0·06)

MMAT check lists

**Reference**	**Screening questions**		**Randomized controlled trials**					**Non-randomized studies**					**Overall score**
	**S1**	**S2**	**2.1**	**2.2**	**2.3**	**2.4**	**2.5**	**3.1**	**3.2**	**3.3**	**3.4**	**3.5**	
Aragonès, 2019, Spain	Yes	Yes	Yes	Yes	Yes	Yes	Yes						***** 100%
Chen, 2010, USA	Yes	Yes						Yes	Yes	Yes	Yes	No	**** 80%
Coventry, 2015, UK	Yes	Yes	Yes	Yes	Yes	No	Yes						**** 80%
Freund, 2016, Germany	Yes	Yes	Yes	Yes	Yes	Yes	No						**** 80%
Jan, 2021, China	Yes	Yes						Yes	Yes	Yes	No	No	**** 80%
Katon, 2004, USA	Yes	Yes	Yes	Yes	Yes	Yes	Yes						***** 100%
Katon, 2010, USA	Yes	Yes	Yes	Yes	Yes	Yes	Yes						***** 100%
Lin, 2012, USA	Yes	Yes	Yes	Yes	Yes	Yes	Yes						***** 100%
Morgan, 2013, Australia	Yes	Yes	No	Yes	Yes	No	No						** 40%
Petersen, 2019, South Africa	Yes	Yes						No	Yes	Yes	Yes	Yes	**** 80%
Petersen, 2021, South Africa	Yes	Yes	Yes	Yes	Yes	Yes	No						**** 80%
Salisbury, 2018, UK	Yes	Yes	Yes	Yes	Yes	Yes	No						**** 80%
Sharpe, 2014, UK	Yes	Yes	Yes	Yes	Yes	Yes	Yes						***** 100%
Towfighi, 2021, USA	Yes	Yes	Yes	Yes	Yes	Yes	Yes						***** 100%
Walker, 2014, UK	Yes	Yes	Yes	Yes	Yes	Yes	Yes						***** 100%
Wolff, 2021, USA	Yes	Yes						No	Yes	Yes	Yes	No	*** 60%
Wood, 2008, USA	Yes	Yes	No	Yes	Yes	No	Yes						*** 60%

S1. Are there clear research questions?

S2. Do the collected data allow to address the research questions?

2.1. Is randomization appropriately performed?

2.2. Are the groups comparable at baseline?

2.3. Are there complete outcome data?

2.4. Are outcome assessors blinded to the intervention provided?
2.5. Did the participants adhere to the assigned intervention?
3.1. Are the participants representative of the target population?
3.2. Are measurements appropriate regarding both the outcome and intervention (or exposure)?
3.3. Are there complete outcome data?
3.4. Are the confounders accounted for in the design and analysis?
3.5. During the study period, is the intervention administered (or exposure occurred) as intended?
