# Supplementary material for: Primary care team and its association with quality of care for people with multimorbidity: a systematic review
Source: BMC Prim Care. 2023 Jan 19;24:20. doi: 10.1186/s12875-023-01968-z (PMC9850572; doi:10.1186/s12875-023-01968-z)
Supplement: Supplementary file 3 — Additional file 3: Table S1. Mechanisms for effective results and ineffective results for primary care teams (PCT). [file 12875_2023_1968_MOESM3_ESM.pdf]

Primary Care Team and its Association with Quality of Care for People with Multimorbidity: A Systematic Review

Supplementary file 3

Table S1. Mechanisms for effective results and ineffective results for primary care teams (PCT)

First author (year) County	Mechanisms for effective results	Mechanisms for ineffective results
Aragonès, 2019, Spain	Not mentioned	- The new intervention was pragmatic and under conditions very close to normal practices.<br>- The control arm received an active therapeutic intervention: the usual treatment.<br>- Patient adherence to one key component of the intervention was poor.<br>- Low adherence of doctors to evidenced-based clinical guidelines.
Chen, 2010, USA	• Stability of team pairings optimized continuity of care for patients and team communication, increasing staff satisfaction.<br>• Health coaches should be carefully selected, well-trained, and observed while interacting with patients, with feedback and protected time.<br>• Active participation and support from departmental leadership<br>• Inclusion of frontline clinic staff members and residents in the planning and implementation of the project.	- Spillover effect, because nursing staff, although acting as health coaches, interacted regularly with all clinic patients.<br>- Incomparable participants between intervention and comparison group.
Coventry, 2015, UK	• A broad range of psychological treatments (behavioral activation, cognitive restructuring, graded exposure, and lifestyle approaches) were tailored to meet the needs of patients.<br>• High integration between mental and physical healthcare providers in primary care.	Not mentioned

Freund, 2016, Germany	• Self-management support, goal setting, and symptom monitoring<br>• Medical assistants, supervised by physicians, were able to assume a new role in chronic care management by means of specific protocols and scripts	- Insufficient intervention intensity<br>- The clustering within physician/medical assistant teams within a practice was not considered.
Jan, 2021, China	• Engaging in data-driven and continuous quality improvement, team-based care can offer higher accessibility to care as well as more effective and efficient delivery by providing care coordination.<br>• Assess quality indicators in a group-wise manner, and reward based on performance of the community healthcare group.	- Lack of close cooperation with heart failure care teams and low-intensity transitional care
Katon, 2004, USA	• Integrated biopsychosocial intervention that focuses on improving both depression and diabetes mellitus management. Focusing only on depression care in not likely to achieve optimal diabetes outcome.<br>• Long-term intervention is needed.	- Spillover effect, because approximately half of the usual care controls in this study received antidepressant treatment
Katon, 2010, USA	• Improve patient outcomes and satisfaction by systematically supporting both patients and the primary care team.<br>• Enhanced patient self-care by nurses with education encompassing self-monitoring, behavioral activation (increase in enjoyable activities), goal setting, and problem-solving improved medication adherence.<br>• Weekly supervision and case reviews by attending physicians and nurses provided timely support for the primary care physician in adjusting medications to achieve specific clinical goals.	- Lack of a control group with the same number of visits.<br>- Spillover effect of the interventions since primary care physicians cared for patients in both the intervention and control groups.
Lin, 2012, USA	• Collaboration among patients, care managers and physicians in setting individualized goals and targets;<br>• Support for patient self-care;<br>• Population-based and systematic monitoring of patient progress;<br>• Timely pharmacotherapy adjustment to achieve treatment goals;<br>• Multidisciplinary consultants for case review with nurse care managers	Not mentioned

Morgan, 2013, Australia	• Use of evidence-based guidelines.<br>• Systematic screening and monitoring of risk factors.<br>• Timetabled recall visits.<br>• New or adjusted roles for team members.<br>• Information support for the clinician.<br>• Enhanced patient self-management.<br>• Identified case manager.<br>• Means of effective communication between all members of the care team.<br>• Audit information for the practice.	- Non-representative sample, because only practices that used clinical software were included in the study<br>- Low patient response rate.<br>- Spillover effect.
Petersen, 2019, South Africa	• Patients correctly identified and referred for further care for their depressive symptoms had a greater chance of having a clinically significant reduction in depressive symptoms.<br>• Change management processes to accompany organizational changes associated with integrated care.<br>• Inclusion of anti-stigma interventions to assist primary care personnel to engage in emotional labor.	- Non-representative sample.<br>- Up-wards bias in detection due to patients' heightened awareness of their potential symptoms.
Petersen, 2021, South Africa	Not mentioned	- Low exposure to the strengthened collaborative care model<br>- Uneven distribution of qualified trained nurses<br>- Mental health not being a priority when improving access and reducing HIV burden was prioritized
Salisbury, 2018, UK	• Support changes in organization, and also increase patients' attitudes on patient-centeredness.<br>• Improvement in quality-of-life measurement tools.	- Insufficient intervention intensity<br>- Non representative sample<br>- Chance imbalance in the primary outcome at baseline;<br>- Possible false positive findings due to the large number of secondary outcomes

Sharpe, 2014, UK	• Intensive: all patients were offered both actively managed antidepressant medication treatment and psychological therapy, and most treatment sessions were face-to-face<br>• Systematically implemented: nurses were trained for 3 months and were regularly supervised during treatment; patient outcomes were monitored regularly<br>• Integrated: integrated with the patients’ cancer and primary care to promote acceptability and adherence.	- Non-representative sample, because the sample was mainly women<br>- Limited generalizability<br>- Unmasked allocation<br>- Unable to establish whether one component of the care was more important than others
Towfighi, 2021, USA	Home visits, clinic visits, and Chronic Disease Self-Management Program workshops were core components	- Insufficient intervention intensity<br>- Spillover effect<br>- Inability to track the care teams' adherence and adaptions to the protocols in real time<br>- Hawthorne effect or participants' interactions with intervention team.
Walker, 2014, UK	• Intensive: all patients were offered both antidepressant medication and psychological treatment<br>• Systematically implemented: nurses were trained until they had proved their competence and patient outcomes were monitored regularly<br>• Rapid response: nurses were trained to quickly engage patients in treatment and the psychiatrists were proactive in providing specific advice to the primary care physicians regarding antidepressant medication<br>• Continuous: sessions were delivered at home and nurses were trained in the use of behavioral activation and problem-solving therapies in the context of deterioration and dying<br>• Integrated: integrated with the patients’ cancer and primary care	- Unmasked allocation<br>- Not able to separate the effectiveness of each individual component<br>- Limited generalizability
Wolff, 2021, USA	Integrating primary care and mental health care	- Difficulty in tailoring program-specific studies to the context, setting and population of the local organizations

		- Varying site-level research capability
Wood, 2008, USA	• Integration of the diagnosis and management of patients with continued preventive care in the same medical facility.<br>• No distinction between symptomatic coronary disease (secondary prevention) and those at high risk (primary prevention) with the same lifestyle and risk factor targets.<br>• Family-entered intervention so that family partners making similar lifestyle changes.<br>• Emphasize lifestyle change: avoidance of tobacco, achievement of a healthy diet, and physical activity were all given equal weighting.<br>• Total risk assessment and management<br>• Simple for implementation: simple equipment was used for supervised exercise sessions.	- Smaller than expected number of patients<br>- Not comparable sample and heterogeneity between pairs of centers<br>- Hawthorne effect<br>- Spillover effect

Table S2. Characteristics of the primary care teams: composition, professional roles, training, communication & supervision

Studies	Type of PCT	Team composition	Professional roles of team members	Intervention training	Communication and supervision
Aragonès, 2019, Spain	Traditional PCT	Care manager: a unique psychologist with expertise in the management of chronic pain, treating physicians	Care manager: follow up patients by telephone, provide therapeutic advice, remind about upcoming appointments, provide cognitive-behavioral psychoeducational program for patients	All physicians assigned to the intervention arm participated in a 90-min session about the intervention details	Care manager delivered and emphasized suggestions to the treating physicians by annotating in the electronic clinical record of each patient<br>Weekly supervision by an expert member of the research team.
Chen, 2010, USA	Traditional PCT	The dyad of a clinician with a medical assistant or health worker (the health coach)	Health coach: help patients manage their own conditions within the context of their daily lives; helped patients build the information, skills, and confidence needed to reach their goals; provide emotional support and practical assistance; call patients between visits to follow-up on action plans and medication adherence and to help patients solve problems and navigate the healthcare system.	Training for collaborative partnership with patients, action plans for healthy behavior change, medication adherence, and an overview of cardiovascular risk factors including diabetes.<br>Requiring active participation through role-plays to develop skills in behavior-change action plan negotiation, medication reconciliation, and patient-centered communication	Not mentioned
Coventry, 2015, UK	Traditional PCT	Case manager (psychological wellbeing practitioners), practice nurse	Psychological wellbeing practitioners: deliver face-to-face sessions of brief psychological therapy; work collaboratively with the patient and practice nurse to check antidepressants adherence, deal with concerns about side	Trained by a multidisciplinary team of psychological therapists, an academic GP with special interests in mental health, and a primary care psychiatrist. Cultural competency	Meetings: a brief collaborative meeting (by telephone or in person) between the patient, the care manager and a practice nurse from the patient's general practice to review patients'

			effects, and help to arrange drug reviews with the general practitioner if necessary.<br>Practice nurse: schedule collaborative meeting between the patient, case manager, and practice nurse	training is delivered by a psychiatrist focusing on translation of guided self-help materials for people of other regions.	progress and plan future care.<br>Supervision: weekly individual supervision by an experienced psychological therapist within their service.
Freund, 2016, Germany	Traditional PCT	Primary care physicians, medical assistants	Primary care physicians and patients negotiate patient-specific goals, with a special emphasis on self-management tasks.<br>Medical assistants: develop specific action plans to achieve these goals together with patients and caregivers; monitor goal achievement and symptom deterioration either face-to-face with patients in the clinic or by telephone using paper-based checklists.	Trained by using a paper-based assessment checklist to reveal patients' needs and resources	Primary care physicians met with medical assistants weekly to review patient progress.<br>Incentivize intervention practice teams by providing $135 per enrolled patient per year to cover staff costs.
Jan, 2021, China	Traditional PCT	5–10 primary care physicians in a single community working in cooperation with a local hospital	Primary care physicians: deliver integrated healthcare services through collaboration within the community healthcare group, focused on preventive care, provide continuous care with 24-hour telephone hotline consultation with hospital doctors and a bidirectional mutual referral network among the primary care clinics and local hospitals, visit inpatient patients in the hospital and participate in the ward round, facilitating further referral back to the primary care clinic.	Not mentioned	Integrated healthcare services provided by primary care physicians were incentivized in addition to a regular fee-for-service payment scheme. On average, each physician was responsible for 550–750 participating patients.

Katon, 2004, USA	Upward collaborative PCT	Depression clinical specialist nurse, primary care physician, a specialist team including a psychiatrist, psychologist, and family physician.	Nurses: problem-solving treatment of depressive disorders, telephone contacts, offer monthly continuation groups if patients with persistent symptoms, review new cases and patients progress	Nurses received an initial 1-week training course on diagnosis and pharmacotherapy and an introduction to problem-solving treatment methods.	Each nurse receives supervision twice a month with a team that included a psychiatrist, psychologist, and family physician to review new cases and patient progress.<br>Nurses interact regularly with the primary care physician.<br>On alternate weeks, nurses review cases by telephone with the psychiatrist supervisor.<br>The psychiatrist supervisor regularly reviews choices and dosages of medication and clinical response and recommend changes.
Katon, 2010, USA	Upward collaborative PCT	Nurses, primary care physicians, psychiatrist, psychologist	Nurses: monitored the patient's progress with respect to management of depression, control of medical disease, and self-care activities, followed patients proactively to provide support for medication adherence, developed a maintenance plan once a patient achieved targeted disease goals, followed patients with telephone calls every 4 weeks.<br>Physicians: supervised and reviewed new cases and patient progress; recommended initial choices and changes in medications	Patients worked collaboratively with nurses and primary care physicians to establish individualized clinical and self-care goals.	Nurses received weekly supervision with a psychiatrist, primary care physician, and psychologist to review new cases and patient progress.<br>The supervising physicians recommended initial choices and changes in medications tailored to the patient's history and clinical response.<br>The nurse communicated recommended medication changes to the primary care physician responsible for medication management.

Lin, 2012, USA	Upward collaborative PCT	A nurse manager, primary care physicians, patients were key members of the team, a psychiatrist and internist or family physician.	Patients, primary care physicians, primary care managers: identify goals or target<br>Patients, care manager, multidisciplinary consultant: monitor progress<br>Physician consultants, care manager: care reviews. weekly multidisciplinary caseload review, formulate treatment adjustments, recommend suggestions to primary care physicians.<br>Care manager: communicate and care coordination; support patients' self-care including motivating and problem-solving, monitoring adherence to medications and life style changes.	Not mentioned	During weekly caseload reviews, physician consultants recommended treatment adjustments to achieve individualized targets for clinical parameters (eg, PHQ-9 score, BP) and ensured accountability for follow-up of the entire caseload.
Morgan, 2013, Australia	Traditional PCT	Nurse, GP	Nurse: leading role in the team, give overall assessment of patients, help patients to develop goals and identify barriers to achieving the goals; consult with patients; refer patients to GP, draft GP Management Plan, coordinate referrals and timetable for follow up	Trained for screening for depression, identification and measurement of physiological risk factors, educating patients in diabetes and heart disease risk reduction	Not mentioned
Petersen, 2019, South Africa	Downward collaborative PCT	Primary care nurses, GPs, monitor nurse, lay counsellors	Nurses: provide supplementary mental health training in basic adult care guidelines<br>GP: oriented to the importance of mental health and upskilled to prescribe antidepressant medications<br>Lay counsellors: deliver individual and group-	Nurses are trained in clinical communication skills to facilitate person-centered care;<br>Lay counsellors are trained and supervised to deliver individual and group-based counselling drawing on	A referral form to monitor nurse referrals to the counsellor

			based counselling drawing on cognitive behavioral therapy techniques for patients with mild to moderate depressive symptoms; strengthen referral pathways for psychosocial counselling.	cognitive behavioral therapy techniques	
Petersen, 2021, South Africa	Upward collaborative PCT	primary care physicians, lay counsellors, primary care nurses, district-based psychologists	The lay counsellors: trained and supervised to provide manualized evidence-based counselling for patients with depressive symptoms under the supervision of a project-employed clinical psychologist.<br>Nurses: receive training about mental health and clinical communications, assess patients at the baseline and 6 months of follow-up<br>Physicians: initiate antidepressant treatment	Supplementary training provided to nurses comprising four additional sessions on mental health content, and four sessions on clinical communication skills.<br>Lay counsellors, with a minimum of 12 years of schooling, are consequently selected, trained and employed to provide service for the duration of the trial.	The lay counsellors provide manualized evidence-based counselling or patients with depressive symptoms under the supervision of a project-employed clinical psychologist. Referral paths that were strengthened through the introduction of lay counselling services.
Salisbury, 2018, UK	Traditional PCT	Nurse, pharmacist, physician	Nurse: identify the health problems most important to the patient, ask about pain, function, and quality of life; screen for depression and dementia; and then address the disease specific care the patient requires.<br>Pharmacist: use the patient’s electronic medical records to review medication, and make recommendations about simplifying and optimizing treatment.<br>Physician: consider the nurse and pharmacist	All practice clinical staff involved in delivering the intervention received two half days of training. Each practice identified a local champion to support implementation.<br>The preliminary findings of nurses were printed as a patient-held agenda to inform the subsequent consultation with the doctor.	All practice clinical staff involved in delivering the intervention received two half days of training, and administrative staff were trained in a separate meeting.<br>Monthly feedback was provided to practices with the extent of completion of reviews, and modest financial incentives (£30) for each completed patient review.

			reviews, discuss treatment adherence, and agree on a collaborative health plan with the patient.		
Sharpe, 2014, UK	Upward collaborative PCT	Cancer nurses, supervising consultation-liaison psychiatrists, patient's oncology team, primary care physician.	Nurse: establish a therapeutic relationship with the patients, provide information about depression and its treatment, deliver brief evidence-based psychological interventions (problem-solving therapy and behavioral activation) and monitor patients' progress<br>Psychiatrists: supervise treatment, aiming to achieve and maintain treatment targets, advise primary care physicians about prescribing antidepressants, and provide direct consultations to patients who are not improving.	Not mentioned	All cases are reviewed weekly in supervision meetings attended by nurses and a psychiatrist.<br>Supervision is informed by an electronic registry that generates graphs of patients' PHQ-9 depression scores over time, provides alerts when these scores do not meet predefined targets, and stores digital video recordings of every treatment session for easy review
Towfighi, 2021, USA	Downward collaborative PCT	A community health worker (trained lay members of the community who serve as patient and community advocates, or "coaches" for disease management), a site principal investigator (PI, a vascular neurologist or cardiologist), and an advanced practice clinician (APC, nurse practitioners or physician assistants)	Community health worker: reinforce and enhance self-management skills; served as a liaison between the patient and the healthcare system; mobilize resources, system support, and friend and family networks to reduce social isolation; and educate participants about vascular risk factors, signs and symptoms of stroke, and activate emergency medical services for stroke symptoms. cellular telephones enabling participants to call the CHWs during business hours with questions and concerns, assisted the participants with transportation arrangements.	The PIs and site PIs trained the APCs to follow evidence-based protocols, teach self-management skills, and educate participants. The APCs also received 80-h training on motivational interviewing	The care team (APC/CHW/PI) at each site held weekly "huddles" to review new participants and jointly developed treatment plans, communicated about participants' progress, and addressed problems. The CHW and APC met more frequently to discuss participants in detail and determined participants' "needs status".

			APC: teach self-management skills, such as BP and glucose monitoring, prescribe medications in the clinic and coordinate care via telephone. A mobile- and web-based care management application is used.		
Walker, 2014, UK	Upward collaborative PCT	Cancer nurses consultation-liaison psychiatrists the patient's primary care physician.	Nurses: established a therapeutic relationship with the patients, provided information about depression and its treatment, delivered brief evidence-based psychological interventions (problem-solving therapy and behavioral activation), and monitored patients' progress. Psychiatrist: supervised treatment, aiming to achieve and maintain targets (PHQ-9 <10 and $\geq 50\%$ drop from baseline), advise primary care physicians about prescribing to ensure rapid initiation and proactive adjustment of antidepressants, and provide direct consultations to patients who are not progressing.	3-month training for patients in the depression care for people with lung cancer program and how to deliver psychological interventions to patients who are physically deteriorating and dying.	All cases were reviewed weekly in supervision meetings attended by nurses and a psychiatrist.
Wolff, 2021, USA	Upward collaborative PCT	Members in the health organizations, mental health providers	These services varied slightly depending on the local model the organization implemented; however, they all included collaboration and coordination between primary care and mental health providers as well as program enhancements, such as transportation or wellness classes, to support participant engagement in the integrated care model.	Not mentioned	Not mentioned

Wood, 2008, USA	Traditional PCT	*Hospital:* two cardiac specialist nurses, a dietitian, a physiotherapist, supported by a lead cardiologist.<br>*General practices:* one cardiac specialist nurse, the family doctors working in the practice.	*Hospitals:* multidisciplinary assessment and management of lifestyle, risk factors, and drug treatment; at 16 weeks, the multidisciplinary team reassess the patients, and a report is sent to their family doctors.<br>*General practices:* assess lifestyle, risk factors, and drug treatment, and assess again at each visit; family doctors responsible for drug treatment; nurses assess the smoking status, health beliefs, set goals to help patients to quit smoking; nurses give advice on food and patterns of eating, and set specific goals for the patients and their families, assess activity patterns, functional capacity etc.<br>nurses: monitor the BP, cholesterol and glucose, review the results with physicians in charge, coordinate workshops for CHD knowledge.	The nurses are trained to address smoking, diet and physical activity, all elements of lifestyle change.	The team is coordinated by the specialist cardiac nurse; the multidisciplinary team send feedback to family doctors at regular time.
